# Impact of vaginal microbiota on the clinical efficacy and long-term recurrence of ketoconazole suppositories in treating severe vulvovaginal candidiasis: a secondary analysis of a multicentre randomised trial

**DOI:** 10.3389/fcimb.2025.1638454

**Published:** 2025-11-24

**Authors:** Lan Mi, Dai Zhang

**Affiliations:** Department of Obstetrics and Gynecology, Peking University First Hospital, Beijing, China

**Keywords:** ketoconazole, severe vulvovaginal candidiasis, treatment, vaginal microbiota, recurrence

## Abstract

**Objective:**

To evaluate whether baseline vaginal-microbiota status influences the clinical cure rate and 30-day mycological recurrence of severe VVC after 6-day intravaginal ketoconazole therapy compared with miconazole nitrate.

**Methods:**

A prospective, randomized, positive drug-controlled, multicenter clinical study was conducted from April 2022 to October 2023 across nine hospitals in China. A total of 253 patients diagnosed with severe vulvovaginal candidiasis were enrolled. The study group received ketoconazole suppositories, while the control group received miconazole nitrate vaginal capsules. Vaginal microbiota results were recorded at enrollment, and follow-up assessments were conducted at 10 and 30 days post-treatment to evaluate the relationship between vaginal microbiota and treatment efficacy and recurrence rates.Vaginal microbiota was categorised by Gram-stain microscopy into (i) normal flora (*Lactobacillus* spp. dominant), (ii) BV-associated abnormal flora (Gram-variable short rods dominant) and (iii) non-BV abnormal flora (other morphotypes).

**Results:**

At enrollment, there were no significant differences between the study and control groups in terms of symptom and sign scores, vaginal dominant bacteria. At the 10-day follow-up, the clinical efficacy rates for patients with normal and abnormal vaginal microbiota were 86.7% and 75.5%, respectively, with a statistically significant difference (P = 0.021). In the study group, the clinical efficacy rates for patients with normal flora, BV-associated abnormal flora, and non-BV-associated abnormal flora were 89.2%, 79.2%, and 78.9%, respectively, with no significant difference (P = 0.264). In the control group, the clinical efficacy rates for patients with normal flora, BV-associated abnormal-flora, and non-BV-associated abnormal flora were 84.1%, 86.7%, and 63.6%, respectively, with a significant difference (P = 0.045).Among the 253 patients, 198 patients who achieved clinical cure and mycological negativity after initial treatment and had mycological follow-up results at 30 days were analyzed. The proportion of mycological positivity at the 30-day follow-up was 14.8% (16/108) in the study group and 26.7% (24/90) in the control group, with a statistically significant difference in recurrence rates between the two groups (P = 0.039). Among the 126 patients with normal dominant bacteria at enrollment,the proportion of mycological positivity at the 30-day follow-up was 12.3% (8/65) in the study group and 26.2% (16/61) in the control group, with a statistically significant difference in recurrence rates (P = 0.047).

**Conclusion:**

The vaginal microbiota, particularly the dominant bacteria, may influence treatment efficacy and recurrence rates. In the treatment of severe vulvovaginal candidiasis, ketoconazole suppositories are not inferior to miconazole nitrate vaginal capsules in clinical efficacy and have a lower long-term recurrence rate.

## Introduction

Vulvovaginal candidiasis (VVC) is a vaginal inflammation caused by Candida infection and is one of the most common gynecological vaginal infections. VVC primarily manifests as pruritus and burning sensation in the vulva and vagina. Clinically, VVC is divided into simple VVC and complex VVC. Severe VVC refers to patients with a symptom and sign score of ≥7 points and is a subtype of complex VVC. Due to its significant symptoms, severe VVC severely affects women’s lives and is therefore a subtype of VVC that is highly focused on in clinical practice. Additionally, recurrent VVC (RVVC), characterized by repeated episodes and chronicity, is challenging to treat and is also a type that receives considerable clinical attention. Currently, the etiology of complex VVC is not fully understood (Sobel and Vempati, 2024). Our previous research has shown that the type of vaginal microbiota significantly affects the prognosis of patients with mixed infections of bacterial vaginosis (BV) and VVC.

Ketoconazole, the first orally active antifungal agent, has been widely used in clinical practice since 1978 to treat both superficial and deep fungal infections due to its high efficacy and broad-spectrum antifungal activity (Gupta and Lyons, 2015). However, because of the severe hepatotoxicity associated with oral formulations, ketoconazole tablets have been withdrawn from the market. In recent years, ketoconazole vaginal suppositories have re-entered clinical practice due to their low systemic absorption and high fungicidal potency.

Last year, we conducted a multicenter, randomized, positive drug-controlled study to evaluate the efficacy and safety of ketoconazole suppositories in treating severe vulvovaginal candidiasis (SVVC) (Mi et al., 2024) This study included patients with both normal and abnormal vaginal microbiota. A new perspective suggests that abnormal vaginal microbiota is also one of the causes of VVC [1]. We re-analyzed the data to assess the impact of abnormal vaginal microbiota on the efficacy of ketoconazole suppositories in treating SVVC.

## 2 Materials and methods

### 2.1 Study overview

This study was conducted from April 2022 to October 2023 by nine hospitals: Peking University First Hospital, Beijing Tsinghua Changgung Hospital, the First Affiliated Hospital of Xi’an Jiaotong University, Xiangya Third Hospital of Central South University, General Hospital of Tianjin Medical University, Peking University Shenzhen Hospital, Women’s Hospital School of Medicine Zhejiang University, Peking University Third Hospital, and the First Affiliated Hospital of Harbin Medical University. The study was a prospective, randomized, positive drug-controlled, multicenter clinical trial involving patients diagnosed with severe VVC. A total of 277 patients were enrolled and randomly assigned in a 1:1 ratio to the study group and the control group using a random number table. The study group consisted of 141 patients who received ketoconazole suppositories, while the control group included 136 patients who were administered miconazole nitrate vaginal capsules. The study was approved by the Ethics Committee of Peking University First Hospital (Ethics approval number: 2021 Research 433) and registered at China Clinical Trial Registry (ChiCTR2200061450).

### 2.2 Study protocol

The eligibility criteria is shown below. Participants were centrally randomized into the study and control groups according to the order of their entry into the study.

#### 2.2.1 Eligibility criteria

• Inclusion:

1. Patients with severe VVC whose symptom/sign score is ≥ 7.
2. Women aged 18–50 years, married or with a history of sexual activity, and not in menstrual period.
3. Women of child-bearing potential who agree to use barrier contraception during the study.
4. Voluntary participation and signed informed consent.

• Exclusion:

1. Pregnant or lactating women.
2. Known contact allergy to any study medication component.
3. Concurrent trichomonas vaginitis or bacterial vaginosis.
4. Systemic antifungal agents within the past 3 months or intravaginal/topical antifungal agents within the past 2 weeks.
5. Any treatment for other vulvovaginal disorders or vaginal douching within the past 2 weeks.
6. Suspected cervical malignancy.
7. Systemic corticosteroids or immunosuppressants within the past 3 months.
8. Participation in another clinical drug trial within the past 3 months.
9. Any other condition that, in the opinion of the investigator, makes the subject unsuitable for enrollment.

#### 2.2.2 Treatment methods

##### 2.2.2.1 Study group

Ketoconazole suppositories (Approval number: National Medicine Standard H20120052, specification: ketoconazole 400 mg, marketing authorization holder: Beijing Jiacheng Biomedical Technology Development Co., Ltd., manufacturer: Zhejiang Shapuaisi Pharmaceutical Co., Ltd.).

Usage: One suppository (400 mg) per night, inserted deep into the vagina while the patient is lying flat, for 6 consecutive days.

##### 2.2.2.2 Control group

Miconazole nitrate vaginal capsules (Approval number: National Medicine Standard J20160028, specification: Miconazole nitrate 400 mg, manufacturer: Xi’an Janssen Pharmaceutical Co., Ltd.).

Usage: One capsule (400 mg) per night, inserted deep into the vagina while the patient is lying flat, for 6 consecutive days.

#### 2.2.3 Efficacy evaluation and follow-up observation indicators

The first follow-up visit was conducted at 10 ± 3 days after the completion of treatment, and the second follow-up visit was conducted at 30 ± 5 days after the completion of treatment. During these follow-up visits, the evaluation of symptoms and signs, microscopic examination of vaginal secretions for mycological assessment, and vaginal microbiota information were performed. All clinical assessments (symptom score, vulvovaginal inspection, adverse-event recording) were conducted by certified gynaecologists trained in the study protocol. The specific scoring criteria are shown in **Table 1** (Cooperative Group of Infectious Disease, C. S. o. O. a. G and Chinese Medical Association, 2012).

**Table 1:** Clinical symptom and sign scoring table.

Symptom/sign	0	1	2	3
Itching	None	Occasional, can be ignored	Noticeable	Persistent, restless
Pain	None	Mild	Moderate	Severe
Congestion, Edema	None	Mild	Moderate	Severe
Excoriations, Fissures, Erosions	None	–	–	Present
Discharge Volume	None	Slightly more than normal	Excessive, no leakage	Excessive, with leakage

##### 2.2.3.1 Primary efficacy endpoint

The primary efficacy endpoint was the clinical efficacy rate at 10 days after treatment completion, defined as the improvement rate of symptom and sign scores ≥67% and negative mycological test results.

The main symptoms and signs included vulvar itching, pain and burning sensation, vulvar erythema and edema, abnormal vaginal discharge, and excoriations, fissures, and erosions. The improvement rate of symptom and sign scores was calculated as follows:


$$Improvement\;rate=\left(\frac{Pre-treatment\;total\;score-Post-treatment\;total\;score}{Pre-treatment\;total\;score}\right)\times 100\%$$

##### 2.2.3.2 Secondary efficacy endpoints

###### 2.2.3.2.1 Clinical efficacy evaluation at 10 and 30 days after treatment completion

- Cure: Symptoms and signs disappear, with an improvement rate of symptom and sign scores ≥67%.
- Effective: Symptoms and signs improve, with an improvement rate of symptom and sign scores ≥30% but <67%.
- Ineffective: No significant improvement or worsening of symptoms and signs, with an overall improvement rate <30%.

###### 2.2.3.2.2 Mycological efficacy evaluation at 10 and 30 days after treatment completion

- Cure: Negative hyphae and blastospores in vaginal secretion smear.
- Uncured: Positive hyphae and/or blastospores in vaginal secretion smear.

##### 2.2.3.3 Vaginal microbiota analysis

**Figure 1:**
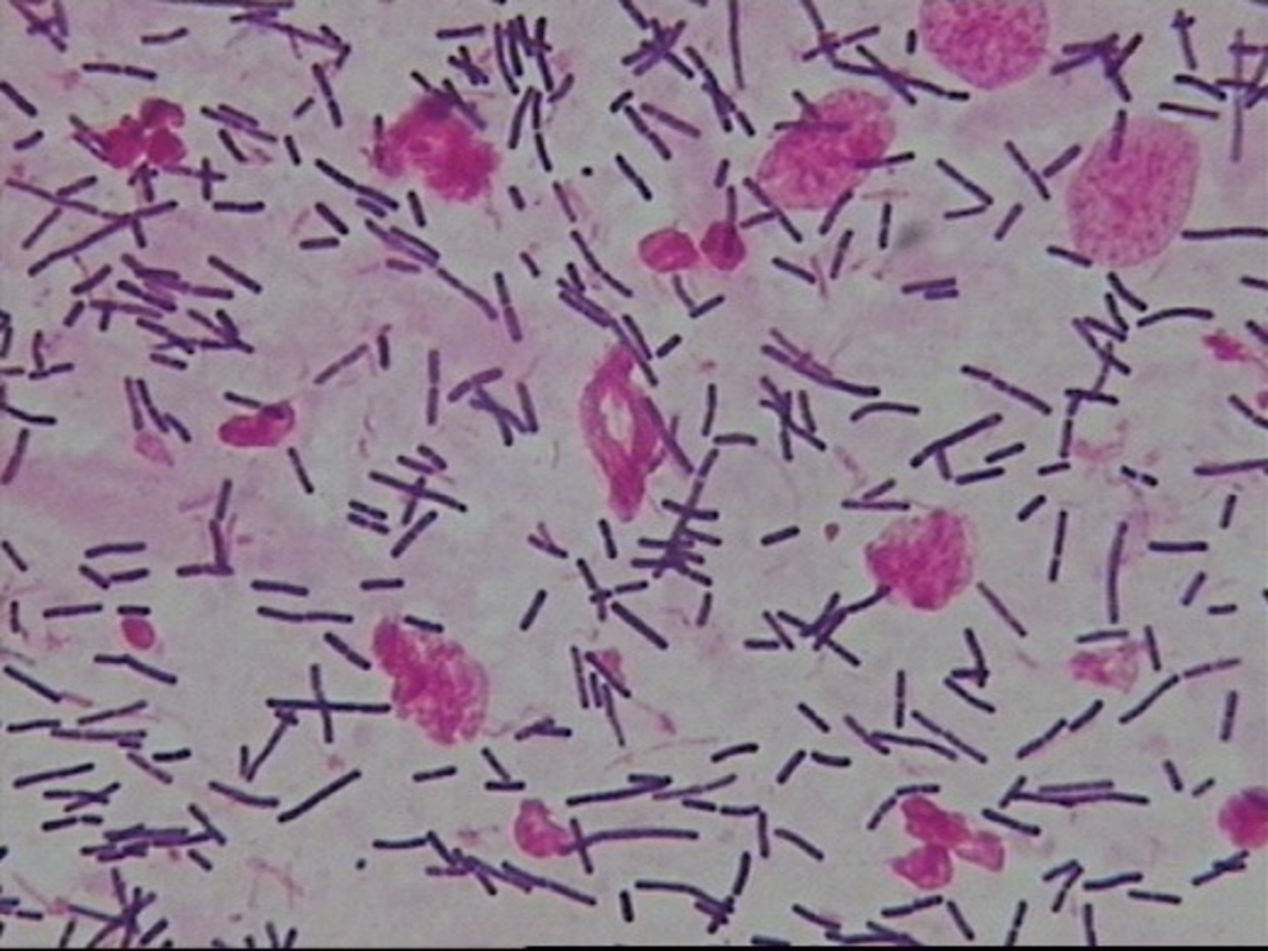
Normal microbiota—Dominant bacteria were Gram-positive large rods (morphologically similar to Lactobacillus spp.).

**Figure 2:**
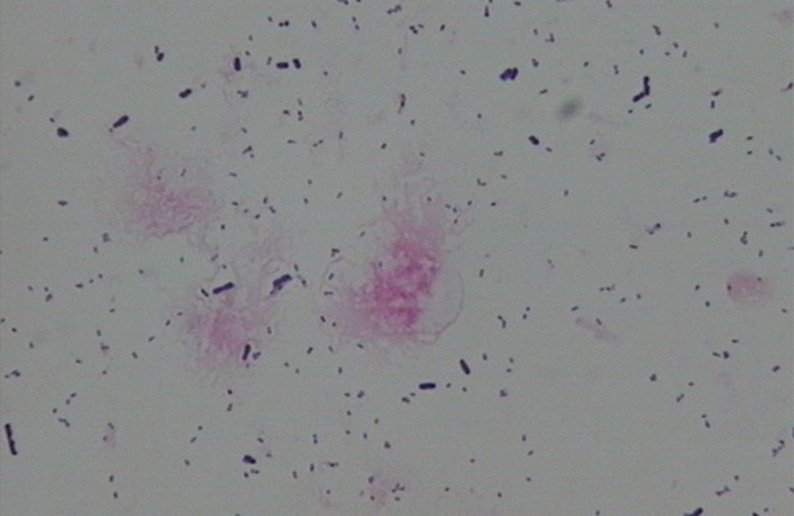
BV-associated abnormal flora—Dominant bacteria were Gram-negative small rods (morphologically similar to Gardnerella vaginalis).

**Figure 3:**
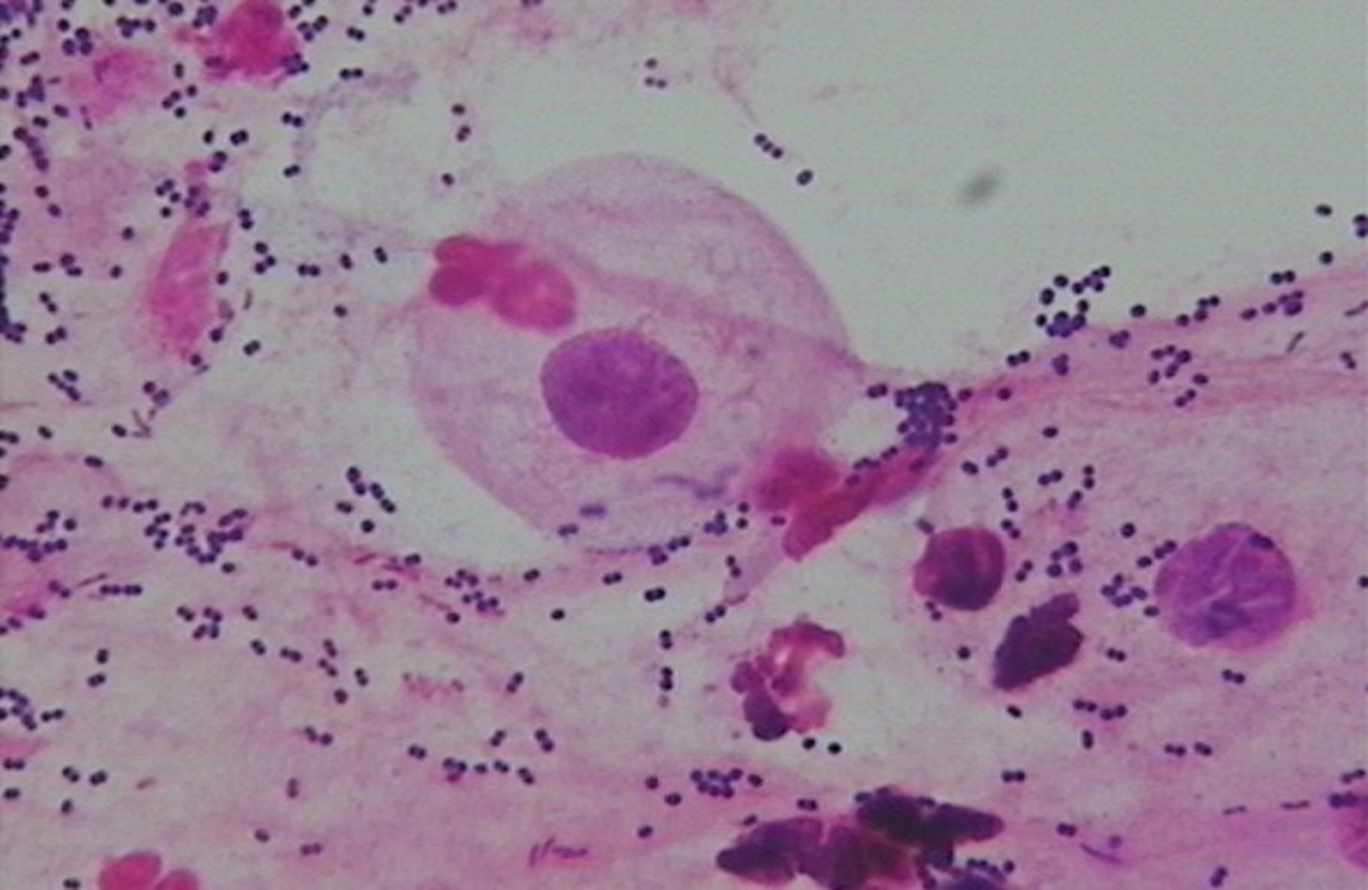
Non-BV-associated abnormal flora—Dominant bacteria were of other types.

Vaginal microbiota was assessed by Gram staining of vaginal secretions under a microscope to determine the dominant bacteria. The most abundant bacteria were identified as the dominant bacteria. The types of dominant bacteria were categorized as follows:

- Normal microbiota: Dominant bacteria were Gram-positive large rods (morphologically similar to *Lactobacillus* spp.) (see **Figure 1**).
- BV-associated abnormal flora: Dominant bacteria were Gram-negative small rods (morphologically similar to Gardnerella vaginalis) (see **Figure 2**).
- Non-BV-associated abnormal flora: Dominant bacteria were of other types (see **Figure 3**).
- The impact of vaginal microbiota types on treatment efficacy was evaluated in conjunction with the aforementioned efficacy endpoints.

### 2.3 Randomisation and blinding

#### 2.3.1 Randomisation method

A stratified block randomisation list was generated by SAS statistical software according to the number of patients at each participating centre and the intended allocation ratio.

#### 2.3.2 Random assignment

Investigators allocated the next sequential randomisation number to each eligible subject in the order of enrolment, recorded this number in the case-report form, and informed the drug dispenser to release the corresponding investigational product according to the randomisation schedule.

This study was not double-blind; however, microbiota assessors and laboratory personnel were blinded to treatment allocation.

### 2.4 Statistical methods

The data were analyzed using SAS software (version 9.4). Efficacy endpoints were analyzed using both Per Protocol Set (PPS) and Full Analysis Set (FAS), while safety endpoints were analyzed using the Safety Set (SS). Categorical data were presented as cases (%), and chi-square tests were used for comparisons. All statistical tests were two-sided, and a P-value of less than 0.05 was considered statistically significant.

### 2.5 Sample size

The primary efficacy endpoint of this trial was the overall response rate at 1 week after treatment. A non-inferiority design was adopted. Based on Phase II data provided by the sponsor, the rate of participants achieving both a ≥ 67% improvement in symptom/sign score and a negative mycological test was 91.15%. The non-inferiority margin was set at –10%, the one-sided significance level α at 0.025, and the power (1 – β) at 0.8. The required sample size was calculated to be 127 participants per group. Allowing for an approximate 20% dropout rate, the total number of participants was set at 318. The sample-size formula is as follows:


$$n_{T}=n_{c}=\frac{{\left(Z_{1-\alpha /2}+Z_{1-\beta }\right)}^{2}\left[P_{c}\left(1-P_{c}\right)+P_{T}\left(1-P_{T}\right)\right]}{{\left(\left|D\right|-△\right)}^{2}}$$

where *P_T_*and *P_C_* are the anticipated event rates in the study and control groups, respectively;

|D| is the absolute difference between the two anticipated rates; $\left|D\right|=\left|P_{T}-P_{C}\right|$ and Δ is the non-inferiority margin.

## 3 Results

### 3.1 Baseline clinical data

A total of 277 patients were enrolled. At enrollment, the mean age of patients in the study group and the control group was 32.22 ± 7.43 years and 31.36 ± 7.19 years, respectively. The mean BMI was 21.72 ± 3.63 kg/m2 and 21.74 ± 3.35 kg/m2, respectively. The baseline symptom and sign scores were 8.19 ± 1.503 in the study group and 8.31 ± 1.594 in the control group. There were no statistically significant differences in these indicators between the two groups (P>0.05).

Of the 277 patients enrolled, 18 were lost to follow-up, 4 violated the protocol, and 2 had drug intake of less than 80% at the 10-day follow-up. A total of 253 patients were included in the clinical and mycological efficacy evaluation. There were no statistically significant differences in the microbiota results between the study and control groups among these 253 patients (P>0.05) (see **Table 2**).

**Table 2:** Comparison of microbiota results between the two groups.

Indicator	n	Study group n (%)	Control group n (%)	X^2^	*P*
pH					
≤4.5	147	76 (55.9)	71 (60.7)	0.596	0.440
>4.5	106	60 (44.1)	46 (39.3)		
Dominant Bacteria					
Normal	143	74 (54.4)	69 (59.0)	1.184	0.553
BV-associated abnormal flora	39	24 (17.6)	15 (12.8)		
Non-BV-associated abnormal flora	71	38 (27.9)	33 (28.2)		
Density					
0	1	1 (0.7)	0	4.768*	0.232
+	1	1 (0.7)	0 (0)		
++	43	28 (20.6)	15 (12.8)		
+++	203	104 (76.5)	99 (84.6)		
++++	5	2 (1.5)	3 (2.6)		
Diversity					
0	1	1 (0.7)	0	1.892*	0.332
+	97	56 (41.2)	41 (35.0)		
++	155	79 (58.1)	76 (65.0)		

*Fisher’s exact test was used for some comparisons due to low expected frequencies.

### 3.2 Relationship between microbiota results and efficacy

Of the 277 patients enrolled, 18 were lost to follow-up, 4 violated the protocol, and 2 had drug intake of less than 80% at the 10-day follow-up. A total of 253 patients were included in the clinical and mycological efficacy evaluation. Among these, 206 patients continued to be followed up and were evaluated for clinical and mycological efficacy at the 30-day follow-up.

#### 3.2.1 Impact of vaginal dominant bacteria on treatment efficacy

At the 10-day follow-up, among the 253 patients evaluated, there were 143 patients with normal vaginal dominant bacteria and 110 patients with abnormal vaginal dominant bacteria. The proportion of abnormal vaginal flora in the study group was 45.6% (62/136), while that in the control group was 41.0% (48/117).The clinical efficacy rates were 86.7% and 75.5%, respectively, showing a statistically significant difference (P = 0.021).

In the study group, the clinical efficacy rates at the 10-day follow-up for patients with normal, BV-associated abnormal, and non-BV-associated abnormal vaginal dominant bacteria were 89.2%, 79.2%, and 78.9%, respectively, with no statistically significant differences (P = 0.264). In the control group, the clinical efficacy rates at the 10-day follow-up for patients with normal, BV-associated abnormal, and non-BV-associated abnormal vaginal dominant bacteria were 84.1%, 86.7%, and 63.6%, respectively, with statistically significant differences (P = 0.045). See **Table 3**. Vaginal pH did not significantly impact treatment efficacy between the study and control groups (P>0.05).

**Table 3:** Impact of vaginal microbiota on treatment efficacy.

Indicator		Study group		X^2^	*P*	Contral group		X^2^	*P*
		Effective	Ineffective			Effective	Ineffective		
pH	≤4.5	66 (86.8)	10 (13.2)	0.688	0.407	55 (77.5)	16 (22.5)	0.147	0.702
	>4.5	49 (81.7)	11 (18.3)			37 (80.4)	9 (19.6)		
Dominant Bacteria	Normal	66 (89.2)	8 (10.8)	2.666	0.264	58 (84.1)	11 (15.9)	6.202	0.045
	BV-associated abnormal flora	19 (79.2)	5 (20.8)			13 (86.7)	2 (13.3)		
	Non-BV-associated abnormal flora	30 (78.9)	8 (21.1)			21 (63.6)	12 (36.4)		

#### 3.2.2 Impact of age on treatment efficacy

There were 175 patients aged 18–35 years old and 78 patients aged 36–49 years old. At the 10-day follow-up, among the 253 patients evaluated, the clinical efficacy rates were 80.0% and 85.9%, respectively, showing no statistically significant difference (P = 0.294). While for patients aged 36–49 years old, the clinical efficacy rates showed significant difference between patients with normal microbiota and abnormal microbiota see **Table 4**.

**Table 4:** Impact of age on treatment efficacy.

Age	Dominant bacteria	Study group		X^2^	*P*	Contral group		X^2^	*P*
		Effective	Ineffective			Effective	Ineffective		
18-35	Normal	42 (84.0)	8 (16.0)	0.613	0.434	40 (83.3)	8 (16.7)	1.344	0.246
	Abnormal	31 (77.5)	9 (22.5)			27 (73.0)	10 (27.0)		
36-49	Normal	24 (100.0)	0		0.045*	18 (85.7)	3 (14.3)		0.197
	abnormal	18 (81.8)	4 (18.2)			7 (63.6)	4 (36.4)		
Total		115 (84.6)	21 (15.4)			92 (78.6)	25 (21.4)		

*Fisher’s exact test was used for some comparisons due to low expected frequencies.

### 3.3 Relationship between microbiota results and recurrence

Recurrence was defined as patients who were clinically cured and had negative mycological results at the 10-day follow-up but had positive mycological results at the 30-day follow-up. Among the 253 patients, 198 patients who achieved clinical cure and mycological negativity after initial treatment and had mycological follow-up results at 30 days were analyzed. The proportion of patients with positive mycological results at the 30-day follow-up was 14.8% (16/108) in the study group and 26.7% (24/90) in the control group. The recurrence rates between the two groups were statistically significant (P = 0.039) as determined by chi-square test. See **Table 5**.

**Table 5:** Comparison of recurrence rates between the two groups.

Mycological	30-day follow-up		Normal dominant bacteria		Abnormal dominant bacteria	
	Positive	Negetive	Positive	Negetive	Positive	Negetive
Study Group	16 (14.8)	92 (85.2)	8 (12.3)	57 (87.7)	8 (17.4)	38 (82.6)
Control Group	24 (26.7)	66 (73.3)	16 (26.2)	45 (73.8)	10 (29.4)	24 (70.6)
Total	40	158	24	102	18	62
χ^2^	4.277		3.955		1.620	
*P*	0.039		0.047		0.203	

To exclude the impact of vaginal microbiota on treatment efficacy, we selected 149 patients with normal dominant bacteria at initial treatment. Among these, 126 patients who achieved mycological negativity after initial treatment and had mycological follow-up results at 30 days were analyzed. The proportion of patients with positive mycological results at the 30-day follow-up was 12.3% (8/65) in the study group and 26.2% (16/61) in the control group. The recurrence rates between the two groups were statistically significant (P = 0.047) as determined by chi-square test. See **Table 5**.

## 4 Discussion

Vulvovaginal candidiasis (VVC) is one of the most common vaginal infections in women, primarily characterized by vulvar itching and increased vaginal discharge. Severe cases, referred to as severe VVC, include severe itching and extensive vulvar erythema, edema, fissures, and erosions, and are considered a type of complex VVC, which is challenging to treat. The U.S. guidelines for the treatment of sexually transmitted infections suggest that severe VVC often responds poorly to common short-course therapies. Therefore, they recommend a 7-14days course of vaginal treatment or two doses of fluconazole 150 mg orally, spaced 72 hours apart (Workowski et al., 2021). The Chinese guidelines for the diagnosis and treatment of vulvovaginal candidiasis (VVC) suggest that the treatment course for severe VVC should be extended compared to that for simple VVC [3]. In this study, the treatment regimens for both the ketoconazole suppositories and miconazole nitrate vaginal capsules were extended based on the standard course for simple VVC. However, a recurrence rate of 20.2% was still observed among the patients. Factors that may influence treatment efficacy include decreased sensitivity of the fungi to the drugs, patient compliance with medication and lifestyle habits. Previous research has indicated that the vaginal microenvironment, particularly the type of microbiota, is also an important factor affecting treatment outcomes. The microbiota detection method is simple and feasible, allowing for rapid assessment of vaginal secretion, which is commonly used in Chinese hospitals for the diagnosis of vaginal infections. Therefore, this study explored the impact of microbiota-related factors on treatment efficacy by analyzing the relationship between vaginal microbiota and treatment outcomes in patients with severe VVC.

### 4.1 Impact of vaginal microbiota on VVC treatment efficacy

In our study, which included 253 patients, the clinical efficacy rates at the 10-day follow-up were 86.7% for patients with normal vaginal dominant bacteria and 75.5% for those with abnormal bacteria. These findings are similar to the results reported by Xiao Z et al (Xiao et al., 2024), suggesting that the type of vaginal microbiota significantly influences treatment outcomes in patients with vulvovaginal candidiasis (VVC).

In the study group, the clinical efficacy rates at the 10-day follow-up were 89.2% for patients with normal dominant bacteria, 79.2% for those with BV-related abnormal bacteria, and 78.9% for those with non-BV-related abnormal bacteria. Although the differences were not statistically significant (P = 0.264), there was a trend towards decreased efficacy in patients with abnormal microbiota.

In the control group, the clinical efficacy rates were 84.1% for patients with normal dominant bacteria, 86.7% for those with BV-related abnormal bacteria, and 63.6% for those with non-BV-related abnormal bacteria. The efficacy rate significantly decreased when the dominant bacteria were non-BV-related abnormal bacteria (P = 0.045).

We stratified participants into 18–35 y (n=175) and 36–49 y (n=78). No significant inter-group differences were detected (P>0.05) in cure and recurrence rates, but the 36–49 y subset with abnormal flora showed a trend toward lower efficacy, which may be attributable to insufficient sample size.

Vaginal microbiota plays a crucial role in both healthy and diseased status. Under normal conditions, the vaginal microbiota is dominated by *Lactobacillus* spp. These beneficial bacteria help maintain the acidic environment of the vagina (pH 3.5-4.5), which in turn inhibits the growth of harmful microorganisms. However, in pathological conditions such as bacterial vaginosis (BV), the vaginal microbiota becomes imbalanced. The number of *Lactobacillus* spp. decreases, while the number of anaerobic bacteria, such as *Gardnerella vaginalis*, increases. This imbalance not only affects vaginal health but may also impact the treatment efficacy of vulvovaginal candidiasis (VVC). 30% of VVC cases involve bacterial co-infections, most commonly with *G. vaginalis*, which increases clinical severity and complicates diagnosis and treatment. The co-occurrence of vaginal infections characterized by the overgrowth of *C. albicans* and *G. vaginalis* at the same time is completely understudied but poses significant therapeutic challenges, as antifungal treatments for VVC do not address the bacterial imbalance in BV, and vice versa (De Seta et al., 2025).

Based on genomic sequencing studies, the vaginal microbiota (VMB) in asymptomatic women can be categorized into five distinct community state types (CSTs). Four of these types are dominated by *Lactobacillus* spp.: CST-I: Dominated by *Lactobacillus crispatus.* CST-II: Dominated by *Lactobacillus gasser*i. CST-III: Dominated by *Lactobacillus iners.* CST-V: Dominated by *Lactobacillus jensenii*. In contrast, CST-IV is characterized by a diverse mixture of facultative and obligate anaerobes, including species of the genera *Gardnerella, Atopobium, Mobiluncus, Prevotella* and other taxa in the order *Clostridiales* (Smith and Ravel, 2017). In our study, normal dominant bacteria were defined as Gram-positive large rods (morphologically similar to *Lactobacillus* spp.), which likely correspond to CSTs I, II, III, and V. Abnormal bacteria related to bacterial vaginosis (BV) and non-BV-related abnormal bacteria may correspond to CST-IV. The former is primarily characterized by Gram-positive or Gram-negative short rods, while the latter includes *cocci* spp. or other miscellaneous bacteria. Under normal physiological conditions, *Lactobacillus* spp. are the dominant bacteria in the vagina. They maintain the balance of the vaginal microbiota by secreting H_2_O_2_, bacteriocins, bacteriocin-like substances, and biosurfactants, which inhibit the growth of pathogenic microorganisms. Moreover, *Lactobacillus* spp. prevent pathogenic microorganisms from adhering to vaginal epithelial cells through mechanisms of competition and competitive exclusion. Research has demonstrated that lactic acid produced by *Lactobacillus* spp. can inhibit a variety of reproductive tract pathogens, including *Neisseria gonorrhoeae*, *Candida* spp., and bacteria associated with BV (O'Hanlon et al., 2013). When the vaginal microbiota is dominated by BV-associated abnormal flora, the abundance of *Lactobacillus* spp. is reduced, leading to an increase in vaginal pH. This change in the vaginal environment promotes fungal colonization and tissue invasion (Huang, 2012; Biswas et al., 2007). When the dominant vaginal flora are non-BV-associated abnormal bacteria, it is hypothesized that these bacteria also have certain interactions with *Candida* spp. Patients with this condition are categorized as having community state type IV-C (CST-IV-C), for which there is limited research available. Postmenopausal women with vaginal atrophy symptoms are often associated with this type (Waetjen et al., 2023). In the control group of this study, when the dominant vaginal flora were non-BV-associated abnormal bacteria, the clinical efficacy rate significantly decreased at the 10-day follow-up. This finding suggests that for this particular population, treatment with miconazole may not be effective, whereas treatment with ketoconazole may be more appropriate.

### 4.2 Impact of vaginal microecology on recurrence of VVC

Among the patients who were cured in the study group and the control group, the proportions of mycological positivity at the 30-day follow-up were 14.8% and 26.7%, respectively. Sobel et al (Sobel et al., 2004). reported that after cessation of treatment, 9.2%, 26.8%, and 57.1% of women experienced recurrence at 6, 9, and 12 months, respectively. The short-term recurrence rate in our study was higher than that reported by Sobel et al., which may be attributed to the fact that our study population consisted of patients with severe VVC, and the treatment did not take into account the influence of vaginal microbiota.

In this study, the recurrence rate was lower in individuals with normal dominant flora in both study and control groups compared to those with abnormal dominant flora, although the difference was not statistically significant. Previous studies on recurrent vulvovaginal candidiasis (RVVC) have shown that the impact of bacteria on recurrence is uncertain and complex, using both next-generation sequencing (NGS) and culture-based methods. Longitudinal NGS analysis revealed that the vaginal bacterial microbiome of RVVC patients lost the menstrual cycle-induced bacterial fluctuations commonly observed in healthy populations (Akimoto-Gunther et al., 2016). Other NGS data (Liu et al., 2013)indicated that the vaginal bacterial microbiome diversity in RVVC patients was similar to or slightly higher than that in healthy populations. Culture-based and microscopic studies have revealed the disruption of both anaerobic and aerobic bacterial microbiota and a reduction in *Lactobacilli* spp. in RVVC patients (Donders et al., 2020; Jian and Su, 2015), suggesting that abnormal microbiota may play a role in the recurrence of VVC.

When analyzing individuals with normal vaginal dominant flora separately, the recurrence rate in the study group (12.3%) was significantly lower than that in the control group (26.2%). This indicates that ketoconazole suppositories are effective in treating severe VVC and are associated with a low long-term recurrence rate. These findings suggest that ketoconazole suppositories are a suitable therapeutic option for severe VVC.

### 4.3 Limitation

Our study has several limitations. First, the analysis of vaginal microbiota was based on Gram staining and light microscopy, which only allows morphological classification at the genus level and lacks species-level resolution; this may have led to misclassification or underdetection of subtle community shifts. This study represents a secondary analysis of data derived from an existing clinical trial primarily designed to evaluate the efficacy and safety of the investigational drug. The further analysis of microbiome data revealed that abnormal flora may influence treatment outcomes, thus the sample size may be underpowered for detecting microbiome-related differences.

In summary, in the treatment of vulvovaginal candidiasis (VVC), the vaginal dominant flora may influence treatment outcomes and recurrence rates. VVC with abnormal vaginal microbiota should also be considered as complex VVC. Excluding the impact of abnormal vaginal microbiota, ketoconazole suppositories demonstrated non-inferior clinical efficacy compared to miconazole nitrate vaginal soft capsules in the treatment of severe VVC, with a lower long-term recurrence rate.

## Data Availability

The raw data supporting the conclusions of this article will be made available by the authors, without undue reservation.
